# Retrospective Analysis of a Modified Organizational Model to Guarantee CT Workflow during the COVID-19 Outbreak in the Tertiary Hospital of Padova, Italy

**DOI:** 10.3390/jcm9093042

**Published:** 2020-09-21

**Authors:** Giacomo Cester, Chiara Giraudo, Francesco Causin, Deris Gianni Boemo, Mariagiulia Anglani, Alfio Capizzi, Giovanni Carretta, Annamaria Cattelan, Diego Cecchin, Vito Cianci, Andrea Crisanti, Giorgio De Conti, Daniele Donato, Luciano Flor, Joseph-Domenico Gabrieli, Marina Munari, Paolo Navalesi, Alberto Ponzoni, Maria Luisa Scapellato, Ivo Tiberio, Andrea Vianello, Roberto Stramare

**Affiliations:** 1 Department of Diagnostic Imaging and Interventional Radiology, Neuroradiology, Padua University Hospital, 35128 Padua, Italy; giacomo.cester@aopd.veneto.it (G.C.); mariagiulia.anglani@aopd.veneto.it (M.A.); josephdomenico.gabrieli@aopd.veneto.it (J.-D.G.); 2 Department of Medicine–DIMED, Institute of Radiology, Padua University Hospital, 35128 Padua, Italy; chiara.giraudo@unipd.it (C.G.); roberto.stramare@unipd.it (R.S.); 3 Department of Directional Hospital Management, Padua University Hospital, 35128 Padova, Italy; derisgianni.boemo@aopd.veneto.it (D.G.B.); alfio.capizzi@aopd.veneto.it (A.C.); giovanni.carretta@aopd.veneto.it (G.C.); daniele.donato@aopd.veneto.it (D.D.); luciano.flor@aopd.veneto.it (L.F.); 4 Department of Internal Medicine, Infectious and Tropical Diseases, Padua University Hospital, 35128 Padua, Italy; annamaria.cattelan@aopd.veneto.it; 5 Department of Medicine–DIMED, Nuclear Medicine Unit, Padua University Hospital, 35128 Padua, Italy; diego.cecchin@unipd.it; 6 ER Unit, Emergency-Urgency Department, Padua University Hospital, 35128 Padua, Italy; vito.cianci@aopd.veneto.it; 7 Department of Molecular Medicine, Microbiology and Virology, Padua University Hospital, 35128 Padua, Italy; andrea.crisanti@unipd.it; 8 Radiology Unit, Department of Diagnostic Imaging and Interventional Radiology, Padua University Hospital, 35128 Padua, Italy; giorgio.deconti@aopd.veneto.it (G.D.C.); alberto.ponzoni@aopd.veneto.it (A.P.); 9 Anesthesia and Intensive Care Unit, Department of Medicine–DIMED, Padua University Hospital, 35128 Padua, Italy; marina.munari@aopd.veneto.it (M.M.); paolo.navalesi@unipd.it (P.N.); 10 Department of Cardiac, Thoracic, Vascular Sciences and Public Health, Preventive Medicine and Risk Assessment, Padua University Hospital, 35128 Padua, Italy; marialuisa.scapellato@unipd.it; 11 Emergency-Urgency Department, Intensive Care Unit, Padua University Hospital, 35128 Padua, Italy; ivo.tiberio@aopd.veneto.it; 12 Respiratory Pathophysiology Unit, Department of Cardiac, Thoracic, Vascular Sciences and Public Health, Padua University Hospital, 35128 Padua, Italy; andrea.vianello.1@unipd.it

**Keywords:** COVID-19, radiology, computed tomography, organizational model, preparedness, outbreak

## Abstract

At the beginning of the severe acute respiratory syndrome coronavirus 2 (SARS-CoV2) outbreak in Italy, the cluster of Vò Euganeo was managed by the University Hospital of Padova. The Department of Diagnostic Imaging (DDI) conceived an organizational approach based on three different pathways for low-risk, high-risk, and confirmed Coronavirus Disease 19 (COVID-19) patients to accomplish three main targets: guarantee a safe pathway for non-COVID-19 patients, ensure health personnel safety, and maintain an efficient workload. Thus, an additional pathway was created with the aid of a trailer-mounted Computed Tomography (CT) scanner devoted to positive patients. We evaluated the performance of our approach from February 21 through April 12 in terms of workload (e.g., number of CT examinations) and safety (COVID-19-positive healthcare workers). There was an average of 72.2 and 17.8 COVID-19 patients per day in wards and the Intensive Care Unit (ICU), respectively. A total of 176 high-risk and positive patients were examined. High Resolution Computed Tomography (HRCT) was one of the most common exams, and 24 pulmonary embolism scans were performed. No in-hospital transmission occurred in the DDI neither among patients nor among health personnel. The weekly number of in-patient CT examinations decreased by 27.4%, and the surgical procedures decreased by 29.5%. Patient screening and dedicated diagnostic pathways allowed the maintenance of high standards of care while working in safety.

## 1. Introduction

**Figure 1:**
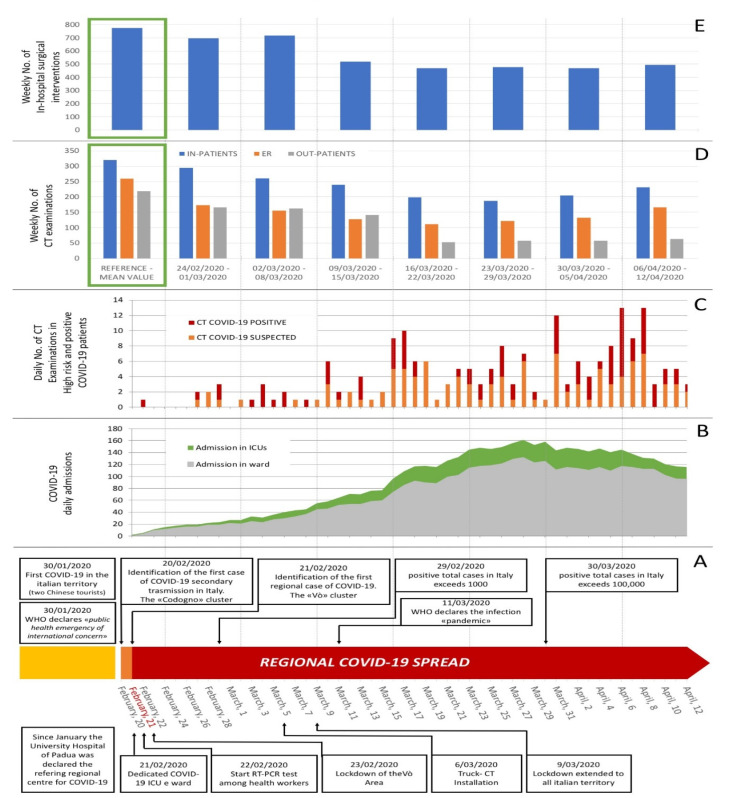
Hospital workload during Phase 1 of Coronavirus Disease 19 (COVID-19) pandemic, showing mean volume per day and week from 21 February through 12 April 2020, death of first patient in Padova area, and identification of the Vò cluster. The reference timeline highlighting the regional and national steps of the pandemic is represented in (**A**). Graph (**B**) shows the daily number of COVID-19 patients admitted to the Intensive Care Unit (ICU) and devoted wards. Graph (**C**) shows the total daily number of CT (Computed Tomography) examinations performed on COVID-19 positive or suspected patients. Graph (**D**) shows the total weekly number of CT acquisitions for in-patients, Emergency Room (ER) and out-patients during Phase 1 of the outbreak from Monday 24 February, and graph (**E**) shows the total weekly number of surgical interventions for in-hospital patients in the same period. Green squares in graph (**D**) and (**E**) identify, as a reference, the mean value of the benchmark performance of CT and surgical activities observed in the four weeks before the outbreak. Both CT scans and surgical interventions for in-patients are shown in blue.

In December 2019, China started to face an outbreak of severe acute respiratory syndrome coronavirus 2 (SARS-COV2), which went on to affect all continents only a few months later [1,2]. In Italy, two clusters were initially identified: the first on the 20th of February 2020 in Codogno, Lombardy, and the second on the 21st of February in the municipality of Vò Euganeo in the Province of Padova [3,4] (Figure 1A). Afterward, both in Italy as a whole and in our highly affected province, the outbreak represented a major challenge for the healthcare system, as it also did worldwide. Various challenges were affronted, regarding many aspects of the pandemic, including the management of patients and healthcare workers. In fact, Coronavirus Disease 19 (COVID-19) requires a tremendous effort for the diagnosis and treatment of infected patients, for the care delivered to negative patients, and for the prevention of disease spread in the general population and among healthcare providers. With this in mind, hospital policies and procedures had to be promptly and efficiently adapted [5,6,7]. The management of the emergency became especially challenging in tertiary centers, such as the University Hospital of Padova, which had to simultaneously face the initial Vò cluster, guarantee care for a catchment area of 950,000 inhabitants, and try not to interrupt the highly specialized care it regularly delivers.

Radiological facilities are among the primary units requiring dedicated management, since radiological imaging can contribute to the diagnosis and monitoring of the disease [8,9,10,11,12]. Chest radiographs and thoracic ultrasound US are easily accessible tools available at the bedside of COVID-19 patients; nevertheless, Computed Tomography (CT) imaging may be also necessary to investigate pulmonary embolism, comorbidities, and/or to carefully evaluate the response to specific treatments [13]. It must also be mentioned that CT examinations require a complex organizational approach in terms of patient transfer among the units, room sanitization, and other measures to preserve healthcare workers’ safety. In fact, a panel of experts led by Maahmud et al. recently stressed the need for preparedness in the Radiology Department [14]. Despite the large body of literature about the current pandemic, to the best of our knowledge, the efficacy of a specific COVID-19 organizational model of radiological units has not been presented yet.

The aim of our study was to analyze the organizational approach applied in the Department of Diagnostic Imaging (DDI) of our tertiary center since the beginning of the local COVID-19 emergency. The model was based on three different pathways for low-risk, high-risk, and confirmed COVID-19 patients to accomplish three main targets: guarantee a safe pathway for non-COVID-19 patients, ensure health personnel safety, and maintain an efficient workload.

## 2. Experimental Section

### 2.1. Diagnostic Department of Imaging

Our Department of Diagnostic Imaging (DDI), which was distributed throughout different buildings in a multi-facility hospital, is composed of one nuclear medicine unit and four radiology units. The radiology units are subdivided into one neuroradiology unit, two general radiology units, and one academic radiology unit. The DDI usually performs more than 46,300 CT examinations per year. Since the identification of the first COVID-19-positive patient (belonging to the initial cluster in Vò) on the 21st of February 2020, structural changes and organizational implementation within the entire DDI have been applied.

Moreover, it must be mentioned that since the 13th of March, regional health authorities obliged hospitals to postpone all elective radiological examinations, except for maternal–pediatric, oncological, and clinically undeferrable conditions.

#### 2.1.1. Usual Radiological Assets

In the main hospital facility, where, among other units, the Emergency Room (ER), the COVID-19 dedicated wards, and Intensive Care Units (ICUs) are located, the following CT scanners are available: one 256 Toshiba Aquilion One (Toshiba Medical Systems, Otawara City, Japan) and one 128 Siemens SOMATOM Definition Edge (Siemens Healthcare, Erlangen, Germany) with a shared control and waiting room, and one Siemens SOMATOM Sensation 64 CT (Siemens Healthcare, Erlangen, Germany) with a separate control room and access path. Three other CT scanners are located in different facilities dedicated to neuroradiology, pediatrics, and orthopedics. The staff of the DDI is composed of 273 healthcare workers, including 45 radiologists and 58 residents.

#### 2.1.2. Structural Implementation

At the beginning of the pandemic spread in Vò, a high-performance mobile trailer-mounted CT scanner (Toshiba Aquilion 128, Toshiba Medical System, Otawara City, Japan) was rented. It was located close to the Emergency Room (ER) and the COVID-dedicated Intensive Care Units (ICUs) of the main building. This option allowed staff to manage infected patients outside the conventional paths, in a completely segregated environment, without any major structural modifications.

#### 2.1.3. Organizational Implementation

**Figure 2:**
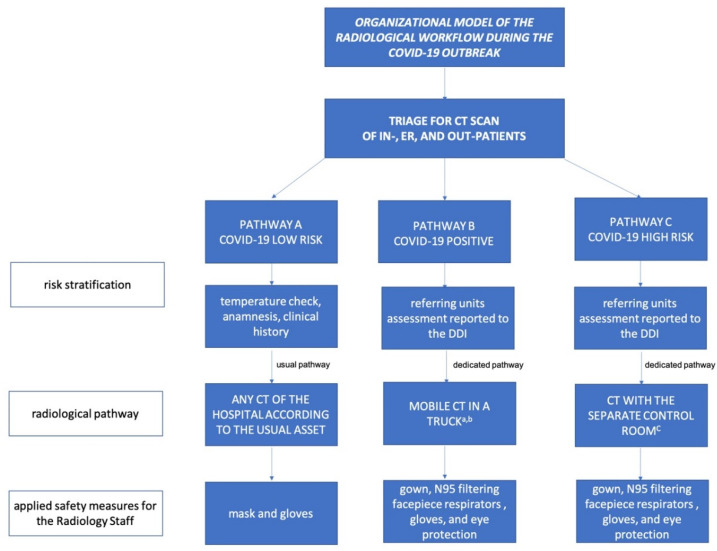
The flowchart represents the protocol applied in the Department of Diagnostic Imaging of the tertiary hospital of Padova to face the COVID-19 emergency. (^a^ rented since the beginning of the pandemic; ^b^ very critical who could have not been transported to the truck were scanned in the closest CT scanner; then, the room was sanitized and locked according to the guidelines; ^c^ then, the CT room was locked and cleaned according to the guidelines. DDI = Department of Diagnostic Imaging).

Aiming to reduce any contamination and guarantee adequate care to other clinical conditions, three separate radiological paths were established (labeled “Pathway A”, “Pathway B”, and “Pathway C”, as described below) for patients in need of a CT examination (Figure 2). In general, for in-patients and patients admitted to the ER, this was done according to a stratification of the COVID-19 risk based on anamnesis, clinical symptoms, and a specific RT-PCR test, in accordance with the guidelines of the World Health Organization [15]. Outpatients with scheduled CT examination were carefully screened prior to the admission to any radiological unit by means of tailored anamnesis (excluding the presence of symptoms and any contact with COVID-19 patients) and check of body temperature (only patients with a temperature <37.5 were admitted). In case of suspect anamnesis or temperature >37.5, outpatients were requested to perform a RT-PCR before admission.

All patients having access to the DDI had to wear a mask during their stay in any area of the DDI.

Pathway A. Patients with low risk of COVID-19 requiring a CT scan:

- Patients admitted to the ER and in-patients: examination was performed in any scanner according to the usual diagnostic pathway.
- Outpatients with scheduled CT examination: examination was performed in any scanner in the hospital according to the usual diagnostic pathway (i.e., CT examinations for neurological diseases are typically performed in the neuroradiology unit).

Pathway B. Patients with positive RT-PCR test results requiring a CT scan:

- Patients admitted to the ER and in-patients: examination was performed using the mobile trailer-mounted CT scanner. Scanner and pathway sanitizations were scheduled twice a day.
- Critical patients who could not be transported to the trailer-mounted CT scanner were examined in any CT scanner, which was then sanitized and halted according to standard precautions associated with airborne pathogens, following national and international guidelines [16,17].

Pathway C. High risk patients without RT-PCR test results, in need of an emergency CT scan:

- In-patients and patients admitted to the ER in need of an emergency CT scan, who had already performed an RT-PCR test but were still waiting for results or who had received an initial negative test, but who had highly suggestive anamnesis and/or symptoms of COVID-19: the CT scan was performed in the main building, in an area with a separate control room and access path. After patient examination, the CT room was sanitized and halted according to the guidelines.

#### 2.1.4. Safety of the Staff

Examination of group B and group C patients: staff were required to wear a gown, boots, N95 filtering facepiece respirators, gloves, and eye protection (goggles or face shield) [18,19]. Examination of group A patients: staff were required to wear a medical mask and gloves. The staff of the DDI was routinely RT-PCR tested every 21 days.

### 2.2. Workload Analysis

#### 2.2.1. Performance of the Applied Model

To describe the efficacy of the applied implementations, we conducted a retrospective evaluation of all CT examinations performed from the 21st of February to the 12th of April 2020 on COVID-19 positive or suspected patients. In particular, the following information was collected: the number of patients that underwent a CT scan, the type of examination (e.g., HRCT, CT for pulmonary embolism, head scan for stroke), and the referring unit (e.g., ER, COVID-19 dedicated ICU or wards). Moreover, to evaluate the overall impact of the outbreak on the DDI, the total weekly number of CT acquisitions performed from Monday 24 February to Sunday 12 April 2020 was collected and compared to the average performance over previous four weeks (from 20 January to 16 February 2020).

The overall number of CT examinations was calculated according to the units in charge of the report (e.g., for one patient who simultaneously underwent a CT scan of the head and the chest, two scans were counted in this study).

#### 2.2.2. Overall Hospital Workload

To provide a comprehensive overview of the impact of the outbreak on the hospital workload, the average number of COVID-19 positive in-patients (both in the ICU and in devoted wards) was assessed for the period from the 21st of February to the 12th of April 2020. In addition, the weekly number of major surgical interventions for in-hospital patients was collected and compared to the weekly average performance as specified in Section 2.2.1. This was done while keeping in mind the fact that deferrable scheduled outpatient and day surgery, as well as open and percutaneous procedures, were postponed due to the national lockdown.

#### 2.2.3. Safety Assessment

To evaluate the impact of the established safety measures, the number of performed swabs as well as the number of COVID-19 positive DDI healthcare workers was calculated. For this variable, the examined interval of time was extended by 21 days to include all DDI healthcare providers who had potentially become infected during the course of the study.

### 2.3. Statistical Analysis

Descriptive statistics regarding the performed examinations were calculated, considering the different types of examinations, the examined anatomical area, the emergency level, and the referring units.

## 3. Results

During the investigated interval of time, there were an average of 72.2 and 17.8 COVID-19-positive patients per day in wards and the ICU, respectively (Figure 1B).

One hundred and seventy-six high-risk and positive COVID-19 patients were examined (110 males and 66 females; mean age ± SD 65.5 ± 17.4 years old), for a total amount of 200 scans, of which 89 were performed for Pathway B (Covid-19-positive patients) (Figure 1C). High-resolution chest CT represented the most common type of requested exam in these two groups of patients, followed by head CTs for neurological symptoms. In 24 patients, CT pulmonary angiograms for pulmonary embolism were performed (Table 1). In the same period, the DDI performed 3337 CT acquisitions for Pathway A (low-risk patients) (Table 2). 

The mean weekly number of overall CT examinations decreased by 40.7% in comparison to the pre-pandemic time interval. On a weekly basis, a decrease in CT scans for outpatients (55.5%), for patients referred by the ER (44.4%), and for in-patients (27.4%) was observed (Figure 1D).

**Figure 3:**
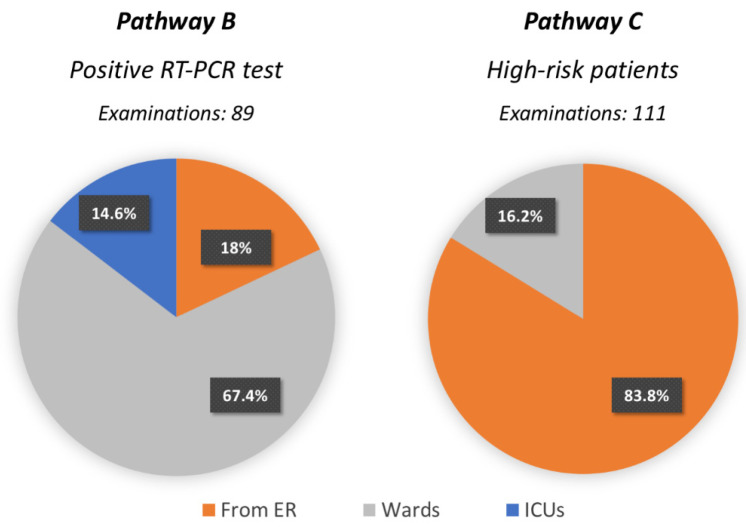
Pie charts representing the units referring for Pathways B and C (high-risk and COVID-19-positive patients) who underwent CT examinations.

Most of the high-risk patients in need of a CT scan were referred by the ER (84%), whereas most of the CTs for COVID-19 positive patients were required by wards (67%) and the ICU (15%) (Figure 3). On average, the weekly number of surgical procedures for in-patients decreased by 29.5% (i.e., 778.75 procedures performed weekly during the pre-pandemic time interval vs. 549.3 during the pandemic, Figure 1E).

To investigate the safety of the applied method, all healthcare providers of the University Hospital of Padova underwent at least one RT-PCR test for COVID-19 during the examined time interval, with an overall number of 24,273 performed tests, of which 175 were positive (0.6%). In the DDI, among 273 health workers, only three technicians tested positive (1%) but they were asymptomatic, and according to their medical history, COVID-19 transmission did not occur at work. Furthermore, no other members of the DDI were infected.

## 4. Discussion

The approach applied in our tertiary center to face the current COVID-19 emergency demonstrates that proper management of the diagnostic pathway guarantees high quality and safety of care both for COVID-19 and non-COVID-19 patients, as well as health personnel safety. Previous studies have already proposed organized workflows in ICU and clinical units [5,6,7,20], which ensured secure work environments.

When it comes to specific work in radiology departments, tailored national and international recommendations [18,19] underlined some essential rules. In particular, it has been emphasized that CT rooms have to be sanitized and halted after examining confirmed COVID-19 patients or patients at high risk of having the disease. The trailer-mounted CT scanner rented for use in our hospital was dedicated exclusively to positive patients, which allowed the delineation of a safe path for these patients, with minor structural changes. Moreover, downtime for sanitizations was not necessary after each patient, as the CT scanner (which was located in a dedicated area) was devoted exclusively to patients with positive RT-PCR test results.

It should certainly be noted that the trailer-mounted CT scanner has some limits. For instance, patients on extracorporeal membrane oxygenation cannot be easily transferred and examined in a truck. In our experience, it occurred once, also because critical COVID-19 patients in the ICU are mainly monitored by chest radiographs at the bedside, as done in previous epidemic such as SARS [21]. Precisely during the SARS outbreak, the crucial role of dedicated devices emerged, as demonstrated by Parmar et al., who used an ICU mobile CT scanner [22]. For the current pandemic, we found the choice of a high-performance trailer-mounted CT scanner especially effective because it allowed high-quality vascular examinations on infected patients. These examinations became crucial since the discovery that COVID-19 may be associated with blood abnormalities, which in turn may cause pulmonary embolism [23,24,25]. In fact, seven out of the 77 CT scans performed using the trailer-mounted scanner were for CT pulmonary angiograms. In addition, this system permits the rapid application of current advanced diagnostic pathways for patients with other symptoms, such as ischemic stroke, which can affect COVID-19 positive patients [12].

An overall decrease in the number of CT scans has been observed since the beginning of the pandemic, which is mostly due to a reduced outpatient count due to the postponement of elective examinations, as decided by the regional healthcare authorities. Despite this, our protocol has proven to be satisfactory in maintaining over 70% of in-patient CT working volumes, in accordance with overall surgical activity.

In addition, our approach also represents a safe model for healthcare workers. Indeed, despite the severe disease spread among healthcare providers worldwide [26], in our DDI, none of the 273 employees contracted the infection at work.

It should not be overlooked that a key element of the favorable outcome of our model is represented by the chance to perform extensive RT-PCR screening to obtain rapid and effective risk stratification. Although Ai and colleagues [27] promoted CT scans as primary tool, we believe that such an approach would not be beneficial in terms of organization if not supported by a dedicated scanner and isolated pathways. Moreover, as reported by Liu et al., not only the rate of positive chest CT findings in positive asymptomatic patients is still unknown, but also the radiation exposure should be taken into account [28].

In a tertiary hospital providing multispecialty care for a large area, dedicated units and pathways are needed to achieve the threefold aim of providing prompt and adequate treatment of infected patients, guaranteeing the regular workflow for patients affected by other diseases, and protecting the safety and health of healthcare workers. In our center, during a global outbreak, this aim has been successfully achieved with the prompt implementation of a dedicated CT scanner, which ensured the organization of three distinct pathways according to COVID-19 risk stratification (i.e., low-risk, high-risk, and positive patients).

Indeed, we strongly believe that our three-path strategy might be suitable not only for a potential second wave of SARS-COV2 infection but will also make us more prepared and resilient in facing future epidemics or pandemics.

Some limitations must certainly be addressed. For instance, our study proposes a model that was found to be very efficient in our tertiary center, but which may be not perfectly suitable for other national or international hospitals. Moreover, spaces for adequate location and dedicated pathways should not be possible in many hospitals, as adjunctive costs should be considered in a complete analysis. In our hospital, the decision to rent a scanner was based on the possibility of having the unit rapidly operational in a specific segregated location during the emergency. This resulted in a minimum impact on the workload of the other scanners of the DDI. Reorganization of the radiological units may need to be adapted according also to the structure of national healthcare systems (e.g., private, public). Studies comparing this approach to other organizational models, and its potential adaptations in different conditions, as well as addressing healthcare policies may provide a better overview and further insights regarding the specific needs of different countries and hospitals.

No specific cost analysis nor clinical information concerning the outcome of the examined patients was included in this study, as it would have exceeded the aim of the project, which was focused on the management of radiological examinations, with a special attention on CT and hospital workflow.

The purpose of this paper is to share our experience and the effort made in order to combine appropriate treatments for infected patients and for other main clinical conditions during the outbreak.

Future studies addressing the clinical outcomes of patients treated under this approach will be also necessary, since this type of data may contribute to the development of an even more accurate model.

In conclusion, in our tertiary center, the combination of a careful screening of patients and dedicated diagnostic pathways allowed us to limit the intra-hospital spread of the infection, to maintain a high standard level of care for all patients, to properly face the emergency, and to work in safety.

## Figures and Tables

**Table 1:** Number of computed tomography examinations performed in our tertiary center for Pathways B and C (high-risk and COVID-19-positive patients) during the COVID-19 pandemic subdivided according to the request pattern.

CT Request Pattern				
PATHWAY B *COVID-19-Positive Patients* *		PATHWAY C *High-Risk Patients* *		
Thorax				
HRCT evaluation	41	HRCT for screening		23
Pulmonary embolism	8	HRCT for diagnosis (negative RT-PCT test)		11
		Pulmonary embolism		16
Head				
Stroke and impaired state of consciousness	9	Stroke and impaired state of consciousness		14
Headache	3	Headache		8
Disequilibrium, vertigo	3	Disorientation		4
Epilepsy	3	Epilepsy		4
		Other		1
Abdomen				
Acute abdomen	1	Acute abdomen		3
Pancreatitis	1	Abdominal occlusion		1
Other	1	Other		2
Vascular				
Aortic dissection	2	Aortic dissection		3
Hemorrhage	5			
Neoplasm				
Follow-up	2	Complications		1
Other Infections				
Exclusion of concomitant infection	1	Exclusion of concomitant infection		3
Trauma				
Head and cervical spine	7	Head and cervical spine		13
Other	2	Polytrauma		1
		Other		

* Risk stratification was performed according to anamnesis, clinical symptoms, and RT-PCR test.

**Table 2:** Number of computed tomography examinations performed in our tertiary center from the 21st of February to the 12th of April 2020 for Pathways A, B, and C (low-risk, COVID-19 patients, and high-risk patients) subdivided according to the origin and the risk of infection.

Origin of Patients	Number of CT Acquisitions							
	Pathway A		Pathway B		Pathway C		Total	
	*Low-Risk*		*Positive*		*High-Risk*			
	(*n*)	(%)	(*n*)	(%)	(*n*)	(%)	(*n*)	(%)
ER	970	29%	16	18%	93	84%	1079	29%
In-patients	1626	49%	73	82%	18	16%	1717	49%
Out-patients	741	22%	0	0%	0	0%	741	22%
	3337	100%	89 ^*^	100%	111	100%	3537	100%

* 12 examinations were not performed in the trailer-mounted CT scanner, 11 of these were performed before the truck became operative, and one was a COVID-19 patient, hospitalized in the ICU, who was on extracorporeal membrane oxygenation and could not be transported to the truck-CT. ER = Emergency Room.

## Abbreviations

COVID-19	Coronavirus Disease 19
RT-PCR	reverse transcription polymerase chain reaction SARS
SARS-CoV-2	severe acute respiratory syndrome coronavirus 2

## References

[1] Lescure F.-X., Bouadma L., Nguyen D., Parisey M., Wicky P.-H., Behillil S., Gaymard A., Bouscambert-Duchamp M., Donati F., Le Hingrat Q. (2020). Clinical and virological data of the first cases of COVID-19 in Europe: A case series. Lancet Infect. Dis..

[2] Tuite A.R., Ng V., Rees E., Fisman D. (2020). Estimation of COVID-19 outbreak size in Italy. Lancet Infect. Dis..

[3] Lavezzo E., Franchin E., Ciavarella C., Cuomo-Dannenburg G., Barzon L., Del Vecchio C., Rossi L., Manganelli R., Loregian A., Navarin N. (2020). Suppression of a SARS-CoV-2 outbreak in the Italian municipality of Vo’. Nature.

[4] Saglietto A., D’Ascenzo F., Zoccai G.B., De Ferrari G.M. (2020). COVID-19 in Europe: The Italian lesson. Lancet.

[5] Grasselli G., Pesenti A., Cecconi M. (2020). Critical Care Utilization for the COVID-19 Outbreak in Lombardy, Italy: Early Experience and Forecast During an Emergency Response. JAMA.

[6] Poston J.T., Patel B.K., Davis A.M. (2020). Management of Critically Ill Adults With COVID-19. JAMA.

[7] Phua J., Weng L., Ling L., Egi M., Lim C.-M., Divatia J.V., Shrestha B.R., Arabi Y.M., Ng J., Gomersall C.D. (2020). Intensive care management of coronavirus disease 2019 (COVID-19): Challenges and recommendations. Lancet Respir. Med..

[8] Pan Y., Guan H. (2020). Imaging changes in patients with 2019-nCov. Eur. Radiol..

[9] Kim H. (2020). Outbreak of novel coronavirus (COVID-19): What is the role of radiologists?. Eur. Radiol..

[10] Fichera G., Stramare R., De Conti G., Motta R., Giraudo C. (2020). It’s not over until it’s over: The chameleonic behavior of COVID-19 over a six-day period. Radiol. Med..

[11] Tosato F., Giraudo C., Pelloso M., Musso G., Piva E., Plebani M. (2020). One disease, different features: COVID-19 laboratory and radiological findings in three Italian patients. Clin. Chem. Lab. Med. CCLM.

[12] Baracchini C., Pieroni A., Viaro F., Cianci V., Cattelan A.M., Tiberio I., Munari M., Causin F. (2020). Acute stroke management pathway during Coronavirus-19 pandemic. Neurol. Sci..

[13] Shi H., Han X., Jiang N., Jiang N., Cao Y., Alwalid O., Gu J., Fan Y., Zheng C. (2020). Radiological findings from 81 patients with COVID- 19 pneumonia in Wuhan, China: A descriptive study. Lancet Infect. Dis..

[14] Mossa-Basha M., Medverd J., Linnau K., Lynch J.B., Wener M.H., Kicska G., Staiger T., Sahani D. (2020). Policies and Guidelines for COVID-19 Preparedness: Experiences from the University of Washington. Radiology.

[15] Clinical Care for Severe Acute Respiratory Infection: Toolkit. World Health Organization. https://apps.who.int/iris/bitstream/handle/10665/331736/WHO-2019-nCoV-SARI_toolkit-2020.1-eng.pdf.

[16] ACR Recommendations for the Use of Chest Radiography and Computed Tomography (CT) for Suspected COVID-19 Infection. https://www.acr.org/Advocacy-and-Economics/ACR-Position-Statements/Recommendations-for-Chest-Radiography-and-CT-for-Suspected-COVID19-Infection.

[17] COVID-19 Situazione in Italia. http://www.salute.gov.it/portale/nuovocoronavirus/dettaglioContenutiNuovoCoronavirus.jsp?area=nuovoCoronavirus&id=5351&lingua=italiano&menu=vuoto.

[18] Operational Considerations for Case Management of COVID-19 in Health Facility and Community. https://apps.who.int/iris/bitstream/handle/10665/331492/WHO-2019-nCoVHCF_operations-2020.1-eng.pdf.

[19] COVID-19 Operatori Sanitari. http://www.salute.gov.it/portale/nuovocoronavirus/dettaglioContenutiNuovoCoronavirus.jsp?lingua=italiano&id=5373&area=nuovoCoronavirus&menu=vuoto.

[20] He Y., Lin Z., Tang D., Yang Y., Wang T., Yang M. (2020). Lancet Strategic plan for management of COVID-19 in paediatric haematology and oncology departments. Lancet Haematol..

[21] Chan S.S., Mak P.S., Shing K.K., Chan P.N., Ng W.H., Rainer T.H. (2005). Chest radiograph screening for severe acute respiratory syndrome in the ED. Am. J. Emerg. Med..

[22] Parmar H.A., Lim T.C., Goh J.S., Tan J.T., Sitoh Y.Y., Hui F. (2004). Providing optimal radiology service in the severe acute respiratory syndrome outbreak: Use of mobile CT. AJR Am. J. Roentgenol..

[23] Xie Y., Wang X., Yang P., Zhang S. (2020). COVID-19 Complicated by Acute Pulmonary Embolism. Radiol. Cardiothorac. Imaging.

[24] Casey K., Iteen A., Nicolini R., Auten J. (2020). COVID-19 pneumonia with hemoptysis: Acute segmental pulmonary emboli associated with novel coronavirus infection. Am. J. Emerg. Med..

[25] Rotzinger D.C., Beigelman-Aubry C., von Garnier C., Qanadli S.D. (2020). Pulmonary embolism in patients with COVID-19: Time to change the paradigm of computed tomography. Thromb. Res..

[26] The Lancet (2020). COVID-19: Protecting health-care workers. Lancet.

[27] Ai T., Yang Z., Hou H., Zhan C., Chen C., Lv W., Tao Q., Sun Z., Xia L. (2020). Correlation of Chest CT and RT-PCR Testing in Coronavirus Disease 2019 (COVID-19) in China: A Report of 1014 Cases. Radiology.

[28] Liu W.H., Wang X.W., Cai Z.Q., Wang X., Huang X.L., Jin Z.G. (2020). Chest CT as a screening tool for COVID-19 in unrelated patients and asymptomatic subjects without contact history is unjustified. Quant. Imaging Med. Surg..

